# Identification of Resistance Genes in Breast Cancer Cells Treated with Fulvestrant and Ribociclib via Retroviral Screening and Integration Site Sequencing

**DOI:** 10.3390/cells15030260

**Published:** 2026-01-29

**Authors:** Zhangzan Huang, Corine Beaufort, Jean Helmijr, Brian Zantboer, Giada Rozema, Camilla Muritti, Julia J. Whien, Anna Uijterwegen, Michele Massimino, John W. M. Martens, Maurice P. H. M. Jansen

**Affiliations:** 1 Department of Medical Oncology, Erasmus MC Cancer Institute, University Medical Centre Rotterdam, Dr. Molewaterplein 40, 3015 GD Rotterdam, The Netherlands; z.huang@erasmusmc.nl (Z.H.); c.beaufort@erasmusmc.nl (C.B.); j.helmijr@erasmusmc.nl (J.H.); b.zantboer@erasmusmc.nl (B.Z.); rozema.giada@gmail.com (G.R.); juliawhien@kpnmail.nl (J.J.W.); a.uijtdewillegen@erasmusmc.nl (A.U.); j.martens@erasmusmc.nl (J.W.M.M.); 2 Department of General Surgery and Medical-Surgical Specialties, University of Catania, 95124 Catania, Italy; 3 Center of Experimental Oncology and Hematology, A.O.U. Policlinico “G. Rodolico—S. Marco”, 95123 Catania, Italy

**Keywords:** breast cancer, retroviral screening, therapy resistance, fulvestrant, CDK4/6 inhibitors, viral integration site sequencing

## Abstract

Around 30% of patients with hormone receptor-positive (HR+) breast cancer acquire resistance to endocrine therapy combined with cyclin-dependent kinase 4/6 inhibitors (CDK4/6i), which are first-line treatments in metastatic settings. Therefore, we aimed to identify loci associated with resistance to endocrine therapy and CDK4/6i; this was achieved using retroviral vectors, which randomly insert gene-disrupting elements into the genome, causing gene expression alterations and potentially leading to therapy resistance. ER-positive ZR75.1 breast cancer cells transduced with retroviral vectors were treated with endocrine (tamoxifen, fulvestrant) or CDK4/6i monotherapies (abemaciclib, palbociclib, ribociclib) or a combination of fulvestrant and ribociclib. DNA was extracted, and virus integration sites (VISs) were characterized according to the detection frequency and read depth using next-generation sequencing (VIS-NGS). Resistance-associated VIS loci were identified when differentially presented in treated samples compared to controls. Well-established tamoxifen resistance genes (*BCAR1*, *BCAR3*, *EGFR*) were detected, enabling the validation of our approach. Thirty-seven VIS loci were associated with resistance to fulvestrant and ribociclib monotherapies. Twenty of these loci were also identified as candidates for resistance to other CDK4/6i and to fulvestrant and ribociclib combination therapy, including *TRPS1* and *TRIM24*—genes that are involved in resistance to endocrine therapy but have not yet been associated with resistance to CDK4/6i. The identification of unique and shared resistance-associated loci highlights the complexity of resistance pathways.

## 1. Introduction

Breast cancer remains the most prevalent malignancy among women worldwide, and its incidence has been slowly increasing since the mid-2000s, largely driven by diagnoses of localized and hormone receptor-positive disease [1]. In 2021, the global burden of breast cancer was estimated at 20.25 million disability-adjusted life years (DALYs) [2]. The disease is highly heterogeneous, both genomically and clinically, and comprises multiple distinct subtypes [3]. Classical immunohistochemistry can be used to identify three major subtypes based on the expression of the estrogen receptor (ER), progesterone receptor (PR), and human epidermal growth factor receptor 2 (HER2).

Advances in breast cancer management have significantly improved patient survival, particularly for those with hormone receptor-positive (HR+)/HER2-negative (HER2-) tumors. This group accounts for approximately 70% of all breast cancer cases [1]. Despite these advances, therapy resistance is inevitable and represents a significant threat to patients, especially in the metastatic setting [4,5].

Tamoxifen, which is a landmark medication for HR+/HER2- breast cancer, has dramatically improved patients’ prognosis over the past few decades [6]. Nonetheless, resistance to tamoxifen and other endocrine treatments has emerged as a major obstacle in clinical practice. Fulvestrant is a selective estrogen receptor degrader (SERD); it was initially used as a monotherapy for advanced ER-positive disease [7,8] and is now approved for use in combination with cyclin-dependent kinase 4/6 (CDK4/6) inhibitors following the MONALEESA-3, PALOMA-3, and MONARCH 2 clinical trials [9,10,11]. Patients benefit from combination therapies, but resistance eventually develops in most cases [4,5].

Novel methodologies such as nanoscale imaging techniques [12] and methods using mechanical biomarkers [13] are currently being utilized alongside conventional functional genetic screening approaches including retrovirus insertion mutagenesis [14], the retroviral transduction of cDNA libraries [15], siRNA/shRNA screens [16,17], and various CRISPR screens [18,19,20] to elucidate the molecular mechanisms underlying therapy resistance. Among traditional techniques, retroviral screening represents a powerful tool for the identification of genes associated with drug resistance. Retroviruses integrate their DNA into the host genome, potentially disrupting or activating adjacent genes and thereby altering the gene expression profile. This distinctive attribute enables researchers to identify genomic regions implicated in resistance. However, conventional retroviral screening methods are often labor-intensive and time-consuming.

To address these challenges regarding retroviral screens, sequencing-based protocols such as viral integration site next-generation sequencing (VIS-NGS) have been developed [21]. VIS-NGS allows the high-throughput and precise mapping of retroviral integration sites within the genome and accelerates the identification of resistance-associated loci, providing comprehensive coverage even when events within the population are rare. The VIS-NGS protocol offers significant advantages over traditional methods, such as reducing the analysis time to just 2 weeks and enabling the detection of low-frequency (1%) integration events, which are difficult to capture with previous approaches.

In the present study, we aimed to address the critical gaps in our understanding of the novel molecular biomarkers underlying therapy resistance in HR+/HER2− breast cancer. We selected candidate genes using the VIS-NGS protocol and exposed retroviral-transduced ZR75.1 breast cancer cells to endocrine therapy and CDK4/6 inhibitors at a sublethal dose. In this way, we sought to reveal novel candidate genes indicating a poor therapy response, deepen our understanding of resistance to endocrine therapy, and guide future investigations toward more effective and personalized therapeutic strategies.

## 2. Materials and Methods

### 2.1. Retroviral Cell Clones and Culture

Parental ER-positive ZR75.1 cells were previously transduced with LN-defective murine retroviruses whose vector backbone included a neomycin resistance cassette, allowing for the selection of successfully transduced cells [14]. The cells were then selected and maintained under standard conditions and included clone IX3, which was generated and checked for its identity two decades previously [14] and stored at −80 °C prior to use. Two independent biological replicates (pLN1, pLN2) were established from the initial thawing and culture of clone IX3 at passage X + 19. After subculturing to passage X + 30, these replicates underwent at least a two-month treatment period and reached passage X + 33, at which point they were used for retroviral screening in this study. Briefly, ZR75.1-transduced cells were cultured in a standard culture medium containing RPMI 1640 medium (Thermo Fisher Scientific, Waltham, MA, USA; Cat. No. 11875-093), 10% heated-inactivated bovine calf serum (BCS, Hyclone, Logan, UT, USA, Cat. No. SH30072.03), 100 pM estrodiol (E2; Med Chem Express, Monmouth Junction, NJ, USA, Cat. No. HY-B0141), and 400 µg/mL of the neomycin analog G418 (Sigma Aldrich, Amsterdam, Netherlands, Cat. No. G8168). All cells were cultured in the standard culture medium for at least two months without and with compounds, with the medium and compounds refreshed weekly and cells split biweekly. All cultures were maintained with 100 U/mL penicillin/streptomycin (Thermo Fisher Scientific, Waltham, MA, USA; Cat. No. 15140122) at 37 °C in a humidified 5% CO_2_ incubator and split until 70–80% confluency was reached. Each treatment condition, including the untreated controls, was implemented in duplicate wells per biological replicate. Untreated cells served as negative controls (NCs) in all analyses.

### 2.2. Compounds and Retroviral Screen Design

Retroviral cell clones were cultured in a standard culture medium with therapeutic compounds. All treatments were administered for at least two months, and cells from both biological replicates were harvested after one month and two or more months of treatment (Supplementary Table S1). The following compounds were used: four-hydroxy-tamoxifen (4-OH-tamoxifen) and fulvestrant were used as endocrine therapy agents, and abemaciclib, palbociclib, and ribociclib were used as CDK4/6 inhibitors. Details of all compounds, including their sources and catalog numbers, are provided in Supplementary Table S2. Compounds were initially dissolved in 100% DMSO (except palbociclib, which was dissolved in 10% DMSO) to obtain stock solutions and then diluted in the culture medium to a compound concentration of 1000 nM. The final DMSO concentration in the medium was below the levels known to affect cell viability (<0.1%), and vehicle control experiments confirmed that DMSO alone did not influence cell growth or morphology. The fixed 1000 nM dose was chosen for all compounds in the 24-well design, because culture confluency and DNA yields resulted in sufficient samples for downstream VIS-NGS analyses. Previous compound experiments in ZR75.1 parental cells without retrovirus yielded an IC50-IC70 below 1000 nM for fulvestrant (IC50 = 296 nM), abemaciclib (684 nM), and the combination of fulvestrant and ribociclib (79 nM), while an IC50 above 1000 nM was observed for tamoxifen (12 µM), ribociclib (8 µM), and palbociclib (1.7 µM). Following the 1000 nM dose, the IC50 doses were evaluated for tamoxifen and fulvestrant. A 10 µM dose was also tested for all compounds but resulted in predominantly low culture confluency (<20%) and insufficient DNA quantities for downstream analyses. The 10 µM dose was only evaluated for fulvestrant, palbociclib, and ribociclib, for one sample each after two months of treatment (Supplementary Table S1).

Retroviral screens for therapy resistance were performed in T25 flasks for 4-OH-tamoxifen and in 24-well plates for all other compounds, and all screens included untreated cells as negative controls (NCs). To avoid variability due to different formats, the seeding densities were adjusted to achieve comparable cell numbers. Each treatment and control group was established with both biological replicates, each seeded in two wells to establish technical replicates, resulting in a minimum of four total replicates per condition. Approximately 70–80% confluency was reached for untreated cells after two weeks of culture, while treated cells were generally less confluent (30–50%). The experimental design included both monotherapy and combination (fulvestrant plus ribociclib) regimens. For each timepoint (one and two or more months of treatment) and biological replicate, cells from two wells were pooled, and DNA was isolated from these cells and processed independently for subsequent VIS-NGS analyses. This resulted in VIS-NGS data for each treatment and from each biological replicate treated for both one month and two or more months. Due to the limited sample numbers, the two timepoints were not evaluated separately but were combined in our downstream explorative VIS-NGS analyses.

### 2.3. DNA Isolation and Quantification

DNA was isolated from cell pellets using the NucleoSpin Tissue Kit (Macherey-Nagel, Duren, Germany, Cat. No. 740952.250), according to the manufacturer’s instructions. Briefly, up to 1 × 10^7^ cells were lysed with Proteinase K and lysis buffers and incubated at 70 °C for 10 min, and DNA was bound to silica columns using ethanol. After washing, DNA was eluted in 100 μL elution buffer. The DNA concentration was measured using the Qubit™ dsDNA HS Assay Kit (Thermo Fisher Scientific, Cat. No. Q32854), with samples diluted 1:200 in Qubit buffer, and fluorescence-based quantification was performed using a Qubit fluorometer (Invitrogen, Waltham, MA, USA, Cat. No. Q32857).

### 2.4. VIS-NGS Protocol

The VIS-NGS analysis was performed as previously described [21]. Briefly, genomic DNA was fragmented using a Covaris S220 focused ultrasonicator (Covaris, Springfield, IL, USA, Cat. No. 500217) to obtain fragments of approximately 1 kb. Fragments were purified with AMPure XP beads (Beckman Coulter, Indianapolis, IN, USA, Cat. No. A63881). End repair and 3′ A-tailing were performed using the NEBNext^®^ Ultra™ End Repair/dA-Tailing Module (New England Biolabs, Ipswich, MA, USA, Cat. No. E7546), followed by linker preparation via the annealing of short and long linker strands (Eurofins Genomics, Wolverhampton, UK, custom oligos). Linkers were ligated to DNA fragments, and a fixed amount of ligated genomic DNA (25 ng) was used for all samples for virus sequence enrichment via PCR. This was performed using forward primers targeted to neomycin and the 3′ LTR region and reverse primers targeted to linkers of the retroviral vector. A nested LTR-PCR using 25 PCR cycles was performed for reamplification. After 300–500 bp fragment size selection and bioanalyzer evaluation, 2 nM sequencing libraries were prepared for all samples using the TruSeq DNA PCR-Free Kit (Illumina, San Diego, CA, USA, Cat. No. 20015962), and pools of libraries were sequenced in paired-end form at 2 × 150 bp on a MiSeq Illumina system with a micro V2 300 cycle flowcell (Illumina, Cat. No. MS-103-1002). Sequence data for both ends were processed using CUTADAPT (v3.4) to remove adapters and low-quality reads and aligned to the hg38 genome using the Burrows–Wheeler Aligner (v0.7.17). Then, VIS mapping and coverage and gene annotation were performed using BEDTOOLS (v2.30.0). CUTADAPT was used to trim adapters and linkers from raw reads, using an error rate of 0.25 and discarding untrimmed reads; the minimum read length was 36 bp. BEDTOOLS was then used to convert alignment files and sort, index, and remove reads with mapping quality values below 20. Reads that mapped within a 1000 bp window were collapsed into a single virus integration site. The median distance from gene loci and integration sites was approximately 19 kb. Reads per VIS locus were normalized to reads per million (RPM) and are presented for 3902 VIS loci detected in this discovery screen (Supplementary Table S3).

### 2.5. Explorative Partial Support Using Single-Cell Transcriptomics

For partial additional support, single-cell RNA-seq data generated for parental and palbociclib-resistant ZR75.1 cells were retrieved from GEO GSE298567 [22]. In total, 2000 parental cells and 1995 palbociclib-resistant cells were evaluated for their expression of the candidate resistance genes identified in our retroviral screen and VIS-NGS analyses. Of the 37 candidate loci, only 21 were recognized according to their gene annotations in the RNA-seq data; the remaining 16 loci were not identified because they had no gene symbols (*n* = 7) or were pseudogenes (*n* = 5), while 4 loci were protein-coding genes without a match in the RNA-seq, including with their aliases. A minimum expression threshold of 1 count per million (CPM; a common threshold for RNA-seq data analysis) in at least one cell was applied to identify cells exhibiting positive expression of a given gene. In the discovery study, for each gene, the fraction of positive cells was calculated for both parental and resistant populations, and statistical differences between these fractions were determined using the chi-squared test, followed by Bonferroni correction for multiple testing.

### 2.6. Statistics

The detection frequency and read depth coverage were obtained from the VIS-NGS sequencing data. Bar charts and volcano plots were generated, and Student’s *t*-tests were performed in Microsoft Excel 365 (Microsoft, Redmond, WA, USA) to obtain the mean read depth coverage at each VIS locus, with a normal distribution between treated and control samples. Using Stata Version 17, Fisher’s exact test was performed to obtain the detection frequency, and a chi-squared test was performed for RNA-seq using the online statistical tool SISA (http://quantitativeskills.com/sisa/; accessed on 4 September 2025). Differences with *p*-values below 0.10 were considered to indicate potential exploratory significance and used for hypothesis-generating findings; those with *p*-values below 0.05 were considered statistically significant. Since this was an explorative study, false discovery rates for VIS-NGS and RNA-seq were not established. As an alternative control for multiple comparisons and false discovery in the VIS-NGS analyses, only VIS loci were selected when detected independently in both biological replicates for each treatment.

## 3. Results

### 3.1. Retroviral Screen and Selection of VIS Loci

**Figure 1:**
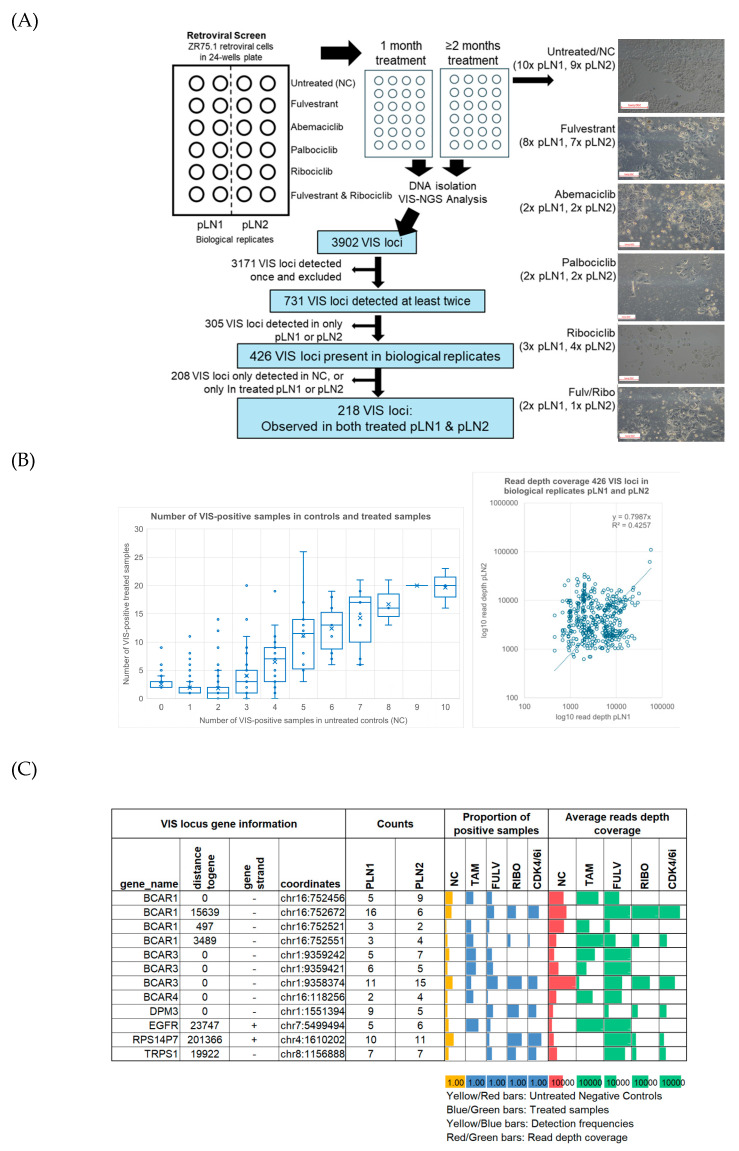
Study overview and overall findings of retroviral screen. (**A**) The workflow for the retroviral screen and VIS-NGS analyses of endocrine- and CDK4/6i-treated retroviral-transduced ZR75.1 cells. The figure provides an overview of untreated and treated cells after at least two months of culture, showing decreased cell numbers and differences in morphology in treated cells compared to untreated cells. The white boxes in the left corners of the photos indicate the size of 500 pixels. The figure also shows the procedures used to identify VIS loci via VIS-NGS and to identify candidate genes for resistance to endocrine and/or CDK4/6i monotherapy. (**B**) Graphs showing 426 loci found in pLN1 and pLN2 and the number of VIS locus-positive samples observed between the treated and control groups; the scatterplot shows the read depth coverage for each locus found in pLN1 and pLN2. Regarding the boxplot, the *X*-axis shows the number of positive samples in the control group, while the *Y*-axis shows the number of positive cases in the treated group. Average numbers are indicated by the X-sign in the boxes and median numbers by horizontal lines. (**C**) VIS-NGS findings (detection frequency, read depth coverage) for seven previously identified tamoxifen resistance genes.

The aim of our explorative retroviral screen was to identify candidate genes for resistance to endocrine therapy and CDK4/6 inhibitors. To achieve this, the retroviral screen was conducted on ZR75.1-transduced cells of clone IX3 in biological replicate cultures using pLN1 and pLN2. The design and workflow of our retroviral screen, as well as examples of untreated and treated retroviral cells, are shown in Figure 1A. In this retroviral screen, a total of 60 samples were evaluated via VIS-NGS, including 19 untreated and 41 treated samples across all conditions (Figure 1A; details provided in Supplementary Table S1). For each treatment, two biological replicates (pLN1 and pLN2) were analyzed. The treated samples comprised single treatments with 4-OH-tamoxifen (*n* = 8), fulvestrant (*n* = 15), ribociclib (*n* = 7), palbociclib (*n* = 4), and abemaciclib (*n* = 4) and a combination treatment with fulvestrant and ribociclib (*n* = 3). Untreated cells served as negative controls in all analyses.

A total of 3902 VIS loci were detected in retroviral samples via VIS-NGS (Figure 1A; Supplementary Table S3). Loci observed only once (*n* = 3171) or not found across both biological replicates—pLN1 and pLN2 (*n* = 305)—were classified as random integration events and excluded from further analyses. The remaining 426 annotated VIS loci were considered of interest because they were detected in at least two independent samples and across both biological replicates. For each of these loci, the associated gene, genomic distance, detection frequency, and median RPM per treatment group were determined to prioritize candidate resistance genes. Identified VIS loci were observed in treated samples, negative controls, or both and at comparable read depth coverage in both pLN1 and pLN2 (Figure 1B).

### 3.2. Detection of Known 4-OH-Tamoxifen Resistance Genes

We identified several VIS loci that have previously been described and validated as 4-OH-tamoxifen resistance genes in a retroviral screen and in recent VIS-NGS analyses (*BCAR1* [14], *BCAR3* [23], *BCAR4* [15,24], *EGFR* [25], *RPS14P7*, *DPM3*, and *TRPS1* [21]; Figure 1C). Multiple VIS loci were identified for two genes, *BCAR1* and *BCAR3*, at different frequencies and read depths and in different subsets of treated samples. For example, in 4-OH-tamoxifen-resistant cells, one VIS locus near *BCAR1* at a distance of 3489 bp was enriched, whereas another locus at a distance of 15,639 bp was depleted, in comparison to the controls. Additionally, a separate locus near or within *BCAR1* was depleted in CDK4/6i-resistant cells relative to untreated samples. Similar patterns were observed for VIS loci associated with *BCAR3*, demonstrating enrichment or depletion depending on their genomic positions and the treatment conditions. These observations indicate that the positions of VIS loci relative to *BCAR1* and *BCAR3* may result in differences in the modulation of gene function, potentially affecting cell growth and resistance to specific therapies. This highlights the importance of considering integration site variability in order to understand resistance mechanisms. All above-reported 4-OH-tamoxifen resistance genes were seen at least twice in both biological replicate samples and were enriched in treated samples, demonstrating that our retroviral screen and VIS-NGS analyses successfully facilitated the characterization of such therapy resistance genes.

### 3.3. Identification of VIS Loci Enriched or Depleted in Therapy-Resistant Cells

To determine whether VIS loci were enriched or depleted in resistant cells compared to untreated controls, we evaluated both the frequency of detection and NGS read depth coverage. The frequency criterion was used to assess whether a VIS locus appeared in a significantly greater or smaller number of treated samples relative to controls and was implemented using Fisher’s exact test. Student’s *t*-test was applied to compare the mean read depth coverage at each VIS locus between treated and control samples, enabling us to detect quantitative differences in locus representation. The rationale behind the use of these complementary tests was to capture both binary presence/absence and more subtle quantitative shifts, thereby increasing the sensitivity of our screen in detecting loci potentially involved in therapy resistance. In this initial discovery phase, we chose a *p*-value threshold of 0.1 for both statistical tests. This relatively relaxed threshold was selected in order to maximize the test’s sensitivity, reducing the risk of overlooking loci that could be relevant to resistance mechanisms. We recognize that this approach may increase the likelihood of false positives; therefore, the loci identified in this phase will be subjected to more stringent validation criteria in subsequent analyses.

From the initial 426 annotated loci, 208 were excluded since they were not detected in either biological replicate—pNL1 or pLN2—across any treatment. The remaining 218 VIS loci, present in both biological replicates relative to controls, were selected for further analysis (Figure 1A; Supplementary Table S4).

Our main aim was to identify genes associated with resistance to endocrine therapies and/or CDK4/6 inhibitors. While all tested compounds targeted distinct pathways, we focused on VIS loci detected in 4-OH-tamoxifen-, fulvestrant-, and ribociclib-resistant cells. Due to the limited sample numbers, the abemaciclib and palbociclib monotherapies, as well as the fulvestrant–ribociclib combination therapy, were not prioritized for separate statistical analyses in this phase. Instead, these groups served as verification cohorts for the VIS loci identified in fulvestrant- and ribociclib-resistant cells. Having established our sample set based on these criteria, statistical analyses were applied to pinpoint VIS loci significantly associated with specific resistance phenotypes.

**Figure 2:**
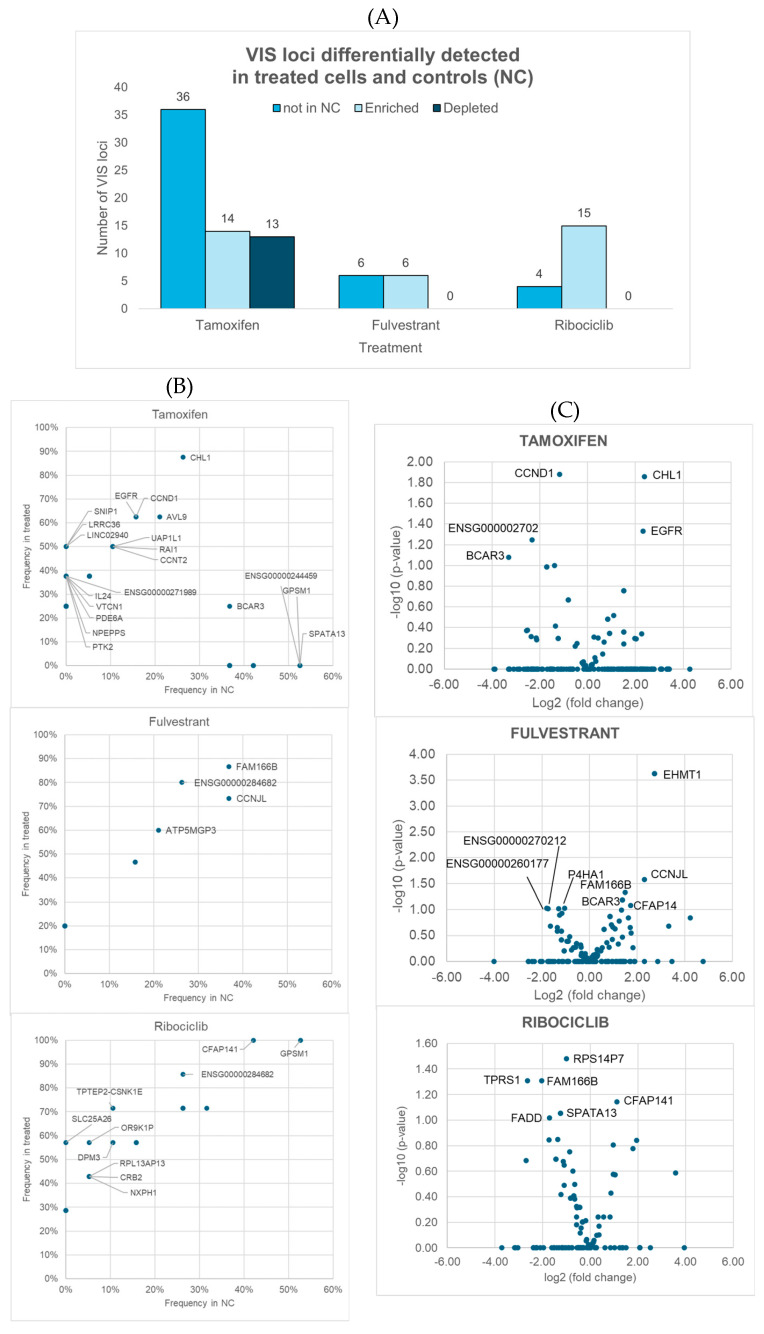
Overview of viral integration site (VIS) loci identified as differentially detected in terms of detection frequency or read depth coverage between samples treated with tamoxifen, fulvestrant, and ribociclib and negative controls (NCs). (**A**) Number of VIS loci found exclusively in treated samples (absent in NCs), as well as those that were either enriched or depleted in treated samples relative to controls when applying *p* < 0.1 as a threshold. (**B**,**C**) Differences between groups: (**B**) shows scatterplots comparing the detection frequencies in controls (*X*-axis) and treated samples (*Y*-axis), while (**C**) presents volcano plots displaying fold changes in read depth coverage (*X*-axis) and statistical significance (*Y*-axis; Student *t*-tests). Both panels show only genes linked to VIS loci with significant differences (*p* < 0.05) between treated samples and controls, although those with *p* < 0.1 were also identified and investigated further.

Fisher’s exact test revealed substantial differences in detection frequency for 89 VIS loci. Of these, 45 were observed exclusively in 4-OH-tamoxifen-, fulvestrant-, or ribociclib-treated cells, suggesting potential treatment-specific integration events that warrant further functional investigation. The other 44 loci were found in both treated and control samples. The distribution of these 89 loci with differential frequencies is summarized per treatment in Figure 2A,B. There were 61 loci unique to 4-OH-tamoxifen, 9 unique to fulvestrant, and 14 unique to ribociclib; moreover, there were five loci across the different treatments (details are shown in Supplementary Table S5). Across all treatment groups, more VIS loci were enriched than depleted compared to the controls (Figure 2A). Scatterplots depicting their detection frequencies (Figure 2B) show the VIS locus genes associated with resistance to 4-OH-tamoxifen, fulvestrant, and ribociclib. In these scatterplots, one dot can represent multiple VIS loci (see the tamoxifen plot for such examples). Only those loci with a *p* < 0.05 in Fisher’s exact test are included in the plots, although those with *p* < 0.1 were also evaluated further. Additionally, Student’s *t*-test revealed significant differences in read depth coverage for 16 VIS loci (Supplementary Table S6), including three (*FAM166B*, *ENSG00000270212*, *CFAP141*) that were detected across several treatments, as shown in the volcano plots (Figure 2C). Notably, *FAM166B* exhibited a high frequency and high read depth coverage in fulvestrant-treated samples but low read depth coverage in ribociclib-treated samples. Four VIS loci were unique to 4-OH-tamoxifen, five were unique to fulvestrant, and four were unique to ribociclib. The plots highlight genes with significantly higher or lower coverage in resistant versus control cells, providing further evidence of their involvement in the therapy response.

Four genes (*BCAR3*, *EGFR*, *CCND1*, *CHL1*) were associated with VIS loci that met the exploratory significance threshold (*p* < 0.1) for both an altered detection frequency and altered read depth coverage in 4-OH-tamoxifen-resistant cells compared to controls (Supplementary Figure S1). Notably, none of these candidate 4-OH-tamoxifen resistance genes were observed or significantly enriched in fulvestrant-treated samples or in any of the CDK4/6 inhibitor-treated samples. Assuming that these genes were not missed by chance, this specificity suggests that the mechanisms of resistance are distinct for 4-OH-tamoxifen compared to fulvestrant or CDK4/6 inhibitors, highlighting that therapeutic strategies targeting these genes may be particularly relevant in overcoming 4-OH-tamoxifen resistance, but they may not be effective for other endocrine therapies. These findings underscore the importance of tailoring therapeutic interventions to the specific resistance mechanisms associated with each type of endocrine therapy. However, *BCAR3* and *EGFR* in the MCF7 cell line induce resistance to both 4-OH-tamoxifen and fulvestrant, which suggests that the cellular context may play a role in the resistance observed.

**Figure 3:**
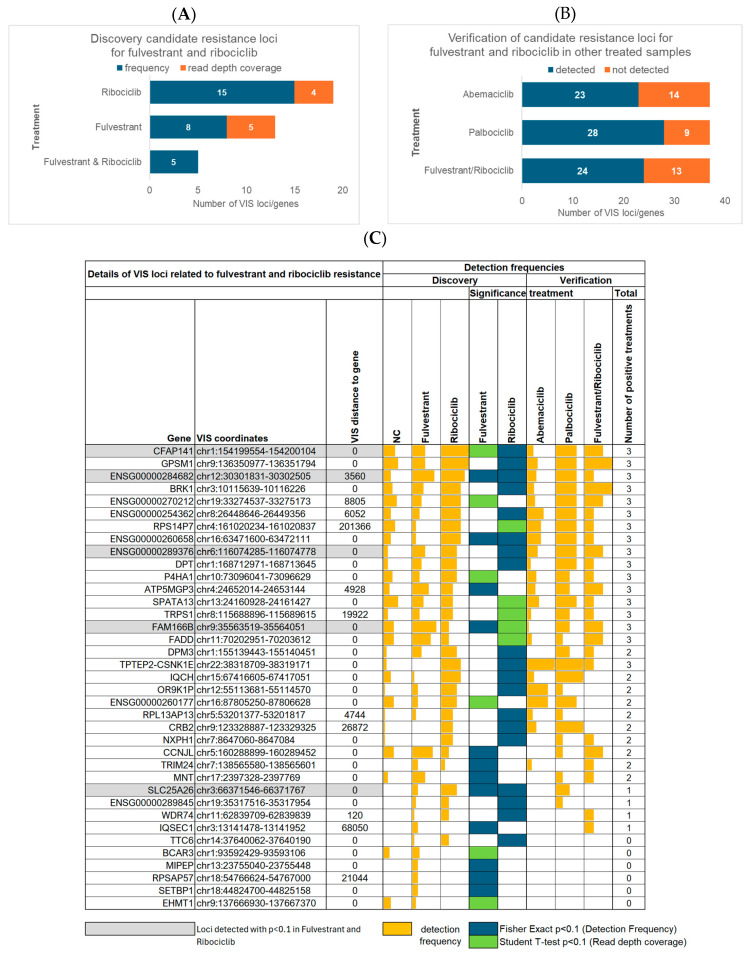
Findings for 37 candidate resistance genes associated with VIS loci that were enriched in fulvestrant- and ribociclib-resistant cells. (**A**) Number of genes identified via differences in detection frequency or read depth coverage in fulvestrant- or ribociclib-treated cells, as well as in both treatments, compared to controls. (**B**) Number of fulvestrant and ribociclib resistance candidates found in samples treated with abemaciclib or palbociclib monotherapy or with fulvestrant and ribociclib combination therapy. (**C**) All 37 candidate genes for fulvestrant and ribociclib resistance. The figure shows their detection frequencies in the discovery cohort of fulvestrant- and ribociclib-treated cells compared to negative controls (NCs). It also indicates whether a gene was of exploratory significance (*p* < 0.1) in terms of the detection frequency according to Fisher’s exact test or in terms of read depth coverage according to Student’s *t*-test between fulvestrant- and ribociclib-treated cells and negative controls (NCs). In addition, the figure presents the detection frequencies of the 37 genes in the verification cohort, which included cells treated with abemaciclib or palbociclib monotherapy, as well as those treated with fulvestrant and ribociclib combination therapy.

In total, 37 genes were identified with significant differences in detection frequency or read depth coverage or both frequency and coverage in cells resistant to fulvestrant or ribociclib or to both treatments compared to the controls (Figure 3A,C). These included 13 loci unique to fulvestrant, 19 unique to ribociclib, and five loci found for both fulvestrant and ribociclib (*SLC25A26*, *FAM166B*, *CFAP141*, *ENSG00000284682*, *ENSG00000289376*) (Figure 3A). An analysis of the resistance mechanisms revealed that only six of these fulvestrant/ribociclib candidate resistance genes (*CFAP141*, *GPSM1*, *RPS14P7*, *SPATA13*, *FADD*, and *ENSG00000260177*) also showed altered detection frequencies in 4-OH-tamoxifen-treated cells compared to controls. However, these genes were all present in the controls but absent in 4-OH-tamoxifen-treated samples, indicating a possible treatment-specific effect.

### 3.4. Verification of Candidate Resistance Genes

To determine the broader relevance of the 37 candidate genes for fulvestrant and/or ribociclib resistance, we verified the presence of these loci in samples treated with abemaciclib or palbociclib monotherapy, as well as with fulvestrant and ribociclib combination treatment. Approximately two thirds of the candidate genes were detected in each of these additional treatment groups (Figure 3B), suggesting that many of the identified loci are not restricted to a single therapeutic agent but may contribute to a wider treatment resistance phenotype. A more detailed analysis (Figure 3C) revealed that 17 genes associated with fulvestrant or ribociclib resistance were consistently detected across all other treatment groups. In contrast, an additional 14 genes were observed in only one or two of the other treatment conditions, reflecting more limited or context-dependent involvement in resistance. Finally, six genes were found to be potentially specific to fulvestrant or ribociclib resistance alone, since they were entirely absent from all samples treated with the other applied therapies. Overall, we identified a set of 20 genes that were detected in cells resistant to fulvestrant and ribociclib mono- and combination therapies. These genes were also found to be upregulated in cells resistant to at least one other CDK4/6 inhibitor, suggesting that they may be involved in shared resistance pathways and warrant functional validation. Interestingly, *TRIM24*, which was recently identified as a therapeutic target for binding to almost all ERα DNA-binding sites [26], was among these genes. In our retroviral screen, *TRIM24* was also detected in cells resistant to abemaciclib monotherapy and the combination of fulvestrant and ribociclib. This suggests that *TRIM24* has a broader role in mediating therapy resistance across multiple treatment types. Moreover, three of the genes that were consistently detected in CDK4/6 inhibitor-treated samples (namely *RPS14P7*, *DPM3*, and *TRPS1*) were identified by our group in the previously obtained 4-OH-tamoxifen-resistant clone VIII-18. However, in the current analysis, these genes were not detected in 4-OH-tamoxifen-treated cells (Figure 1C). This suggests that limitations in terms of screen coverage and selection bottlenecks played a role, whereby screening only allowed enrichment for a limited number of clones, and these factors could affect the representation of certain loci. The coordinates of the VIS loci for these three genes identified via VIS-NGS were similar but not identical between the retroviral screen and clone VIII-18, suggesting independent events (Supplementary Table S7).

Additionally, our findings indicate that a substantial proportion of the identified candidate genes for fulvestrant and ribociclib resistance are involved in common resistance pathways across multiple CDK4/6 inhibitor regimens. This overlap highlights the need to validate these loci and to determine whether they could serve as broader biomarkers or therapeutic targets to overcome resistance in clinical settings; in this way, they could provide a strong foundation for future validation and mechanistic studies and translational applications.

### 3.5. Single-Cell Transcriptomics of Candidate Resistance Genes for Preliminary Validation

**Figure 4:**
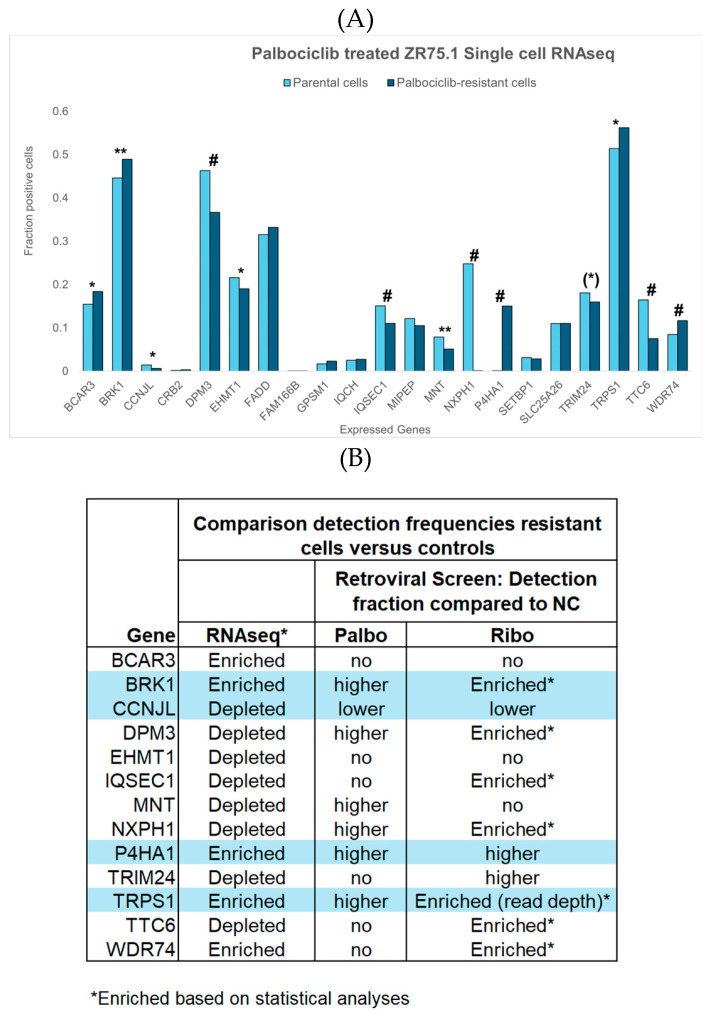
Single-cell RNA-seq results from parental and palbociclib-resistant ZR75.1 cells, as determined recently by Migliaccio et al. [22]. Of our 37 candidate resistance genes, data were available for 21. (**A**) Fractions of parental cells (light blue bars) and palbociclib-resistant cells (dark blue bars) expressing each gene of interest, as well as their significance levels (* *p* < 0.05, ** *p* < 0.001, # *p* < 0.001 after Bonferroni correction). Thirteen genes exhibited differences in the proportion of positive cells between parental and resistant populations, as determined via the chi-squared test. Notably, *TRIM24* showed a trend toward significance (^(^*^)^ *p* = 0.076), while *BCAR3*, *CCNL*, *EHMT*, and *TRPS1* reached the conventional threshold for statistical significance (*p* < 0.05), and *BRK1*, *DPM3*, *IQSEC2*, *MNT*, *NXPH1*, *P4HA1*, *TTC6*, and *WDR74* were highly significant (*p* < 0.001); all remained significant after Bonferroni correction for multiple testing, except *BRK1* and *MNT*. (**B**) Comparison of the most significant findings of the RNA-seq analysis with those of our retroviral screen and VIS-NGS analyses for palbociclib and ribociclib. The four genes in blue boxes/areas demonstrated consistent results in both RNA-seq and the retroviral screen, with three genes (*BRK1*, *P4HA1*, *TRPS1*) enriched in resistant cells and one gene (*CCNJL*) depleted in resistant cells relative to controls. Three genes (*DPM3*, *MNT*, *NXPH1*) displayed a higher detection fraction for palbociclib than in controls in our retroviral screen but were depleted in resistant cells according to RNA-seq. The remaining six genes were not detected in our retroviral screen for palbociclib.

Since VIS loci may result in gene expression changes, the expression of candidate resistance genes in relation to CDK4/6 inhibitor resistance was evaluated in a preliminary single-cell RNA-seq analysis of parental and palbociclib-resistant ZR75.1 cells, recently described in a single-cell transcriptomics study of breast cancer cell lines [22]. Thirteen of the 21 candidate genes for which RNA-seq data were available showed a significant difference in the proportion of positive cells between parental and resistant populations, providing initial confirmation. Differences were observed for the genes *BRK1*, *DPM3*, *IQSEC2*, *MNT*, *NXPH1*, *P4HA1*, *TTC6*, and *WDR74* (*p* < 0.001) and the genes *BCAR3*, *CCNJL*, *EHMT*, and *TRPS1* (*p* < 0.05), as well as a trend for *TRIM24* (*p* = 0.076). After Bonferroni correction for multiple testing, only *DPM3*, *IQSEC1*, *NXPH1*, *P4HA1*, *TTC6*, and *WDR74* remained significant. The RNA-seq results were consistent with those of our retroviral screen and VIS-NGS analyses of palbociclib and ribociclib regarding four genes (*BRK1*, *P4HA1*, *TRPS1*, *CCNJL*; see Figure 4B). In our retroviral screen, three genes (*DPM3*, *MNT*, *NXPH1*) displayed a higher detection fraction for palbociclib than for the controls, while they were depleted in resistant cells according to RNA-seq. The remaining six genes were not detected in our retroviral screen for palbociclib. It should be noted that the validation described here is partial and exploratory, as not all genes were evaluable and only a subset exhibited directionally consistent changes.

## 4. Discussion

Resistance to endocrine therapy remains a major challenge in HR+/HER2- breast cancer [27], undermining treatment effectiveness and leading to delayed clinical decisions, increased costs, and missed opportunities for optimal intervention—ultimately contributing to poor prognoses. Identifying predictive biomarkers that could be used to guide treatment selection and optimize patient outcomes is an important goal [28]. Such biomarkers enable early recognition of non-responders, allowing for timely adjustments to therapy and supporting the development of more tailored treatment approaches. Additionally, elucidating the functional roles of biomarkers enhances our understanding of the molecular mechanisms driving endocrine resistance and may reveal novel therapeutic targets [29]. For example, the *PIK3CA* mutation indicates a poor response to endocrine therapy [30], but PIK3CA inhibitors can overcome this resistance [31]. Nonetheless, these opportunities are contingent upon further functional and clinical validation.

In this study, we combined a retroviral screen with sequencing to identify candidate genes associated with treatment resistance across biological and technical replicates. By integrating the treatment-induced enrichment of the detection frequency and read depth coverage, we prioritized VIS loci with potential functional relevance to the resistant phenotype for fulvestrant and/or ribociclib monotherapy (37 genes) and for tamoxifen therapy (four genes). Notably, the selected genes were consistently detected across biological and technical replicates and demonstrated increased abundance in the treated groups compared to untreated controls, suggesting roles in therapy adaptation and resistance mechanisms. Among the candidate resistance genes, three genes (*BCAR3*, *EGFR*, *CCND1*) have already been functionally implicated in resistance to endocrine therapy [32,33,34], highlighting that our approach—combining a retroviral screen with VIS-NGS and selection criteria—is well suited to the identification of both established and potentially novel mediators of treatment resistance. Importantly, a subset of candidate genes has not previously been linked to therapy adaptation, underscoring the novelty and potential significance of our findings and expanding the landscape of genes that may contribute to resistance mechanisms.

*RPS14P7* and *TRPS1* were detected via VIS-NGS in fulvestrant-treated cells and in all three groups of CDK4/6 inhibitor-treated cells, but not in tamoxifen-treated cells. However, these genes were previously identified in tamoxifen-resistant retroviral clone VIII-18 using VIS-NGS [21]. These discrepancies may be attributable to differences in the experimental design (e.g., the size of the library screened, type selection, cell culture conditions, etc.).

Transcriptional repressor GATA binding 1 (*TRPS1*), which we have identified as a candidate resistance gene, has also been described as a tamoxifen resistance gene, with roles in regulating estrogen receptor binding [35], progesterone signaling [36], YAP/TEAD-dependent transcription [37], and luminal progenitor maintenance [38] in breast cancer cells. Our retroviral screen also identified another gene, *TRIM24*, which has emerged as an estrogen receptor co-factor and plays a leading role in resistance to tamoxifen [26]. In our research, *TRPS1* and *TRIM24* have been implicated in broader biological functions. *TRPS1* is linked to resistance to fulvestrant and CDK4/6 inhibitors, while *TRIM24* is associated with resistance to ribociclib and abemaciclib monotherapies, as well as fulvestrant and ribociclib combination therapy. Previous studies have highlighted these two genes as potential therapeutic targets. Our findings confirm that *TRPS1* and *TRIM24* are strong candidates for future studies aimed at investigating mechanisms of resistance and exploring new targeted therapeutic strategies.

Finally, the expression levels of the candidate resistance genes identified in our retroviral screen were verified using publicly available single-cell transcriptomics data from ZR75.1 parental cells and palbociclib-resistant cells. Despite investigating genes at different levels (virus integration sites in DNA versus RNA expression) in relation to therapy resistance, the consistent results obtained across both methodologies support the notion of the involvement of these genes in resistance to CDK4/6 inhibitors. Contrasting expression patterns between VIS and RNA-seq were observed for several genes, likely due to factors such as disruptive versus activating mutations, clonal heterogeneity, and varying drug regimens or treatment contexts. These variables contribute to divergent gene expression profiles, underscoring the complexity involved in interpreting genomic data in cancer research. The differences between the RNA-seq and retroviral screen results may also have arisen from methodological and detection threshold variations, highlighting the importance of using complementary approaches to validate the findings. It is important to note that these analyses provide initial, partial support; further analyses, such as the functional validation of these genes in resistance models, are needed to confirm their roles in CDK4/6 inhibitor resistance and to potentially identify new therapeutic targets.

Generally, the VIS-NGS method demonstrated considerable sensitivity in detecting VIS loci, even with the use of low-yield samples obtained from 24-well plates. For example, our analyses successfully identified resistance-associated loci in both biological replicates across treatments; we also detected low-frequency integration events that would likely have been missed under less sensitive approaches. The consistent detection of specific loci, such as *RPS14P7*, *DPM3*, and *TRPS1*, in resistant cell populations [21] supports the reliability of the technique and underscores its utility for studies with limited input material.

A notable limitation of this pilot study, which aimed to provide proof of principle, lies in the comparatively small quantity of cells used for screening (i.e., 1–2 × 10^4^ cells in one well of a 24-well plate). The limited yield of resistant cells and thus genomic DNA (50–100 ng) may have reduced the complexity of the library and introduced potential biases in our findings. The limited number of samples available for abemaciclib and palbociclib monotherapies, as well as fulvestrant and ribociclib combination therapy, also restricted the statistical power of the analyses in these groups. In the future, expanding the sample size and improving the DNA quality will minimize the risk of missing rare integration events and strengthen the statistical power and generalizability of the findings. In addition, multiple loci detected in a single clone and lenient selection thresholds raised the possibility of false positives. As this was an exploratory study, we did not correct for multiple comparisons in our VIS-NGS analyses, and we used a relatively lenient cutoff of *p* < 0.1. This could have led to the overestimation of the significance of our findings, which may not be biologically relevant.

In conclusion, we identified a list of resistance-associated genes, whose consistency with well-known genes supports the validity of our findings. While further validation is needed to confirm the presence of these candidates, our results demonstrate the sensitivity of the applied method, even with limited samples. Despite these promising outcomes, larger sample sets and further functional validation are necessary to fully establish the roles of these genes in resistance. Future research should also focus on validating these genes in preclinical and clinical models and optimizing our detection method for broader practical use. These efforts may eventually contribute to advancing personalized treatment strategies in oncology.

## Data Availability

The VIS-NGS fastq files (Supplementary Materials) and the data files generated in this study are available upon request by emailing the corresponding author (m.p.h.m.jansen@erasmusmc.nl).

## Supplementary Materials

The following supporting information can be downloaded at https://www.mdpi.com/article/10.3390/cells15030260/s1: Figure S1: Findings for 4 candidate tamoxifen resistance genes; Table S1: Details of retroviral screen samples and VIS-NGS, Table S2: Compound details, Table S3: Details of 3902 VIS loci detected in retroviral screen, Table S4: Details and Analysis summary of 218 VIS loci detected in both biological replicates, Table S5: Candidate resistance VIS loci (*n* = 89) for tamoxifen, fulvestrant, and/or ribociclib with differential detection frequencies compared to controls, Table S6: Candidate resistance VIS loci (*n* = 16) for tamoxifen, fulvestrant, and/or ribociclib with differential read coverage compared to controls, Table S7: VIS-locus coordinates of TRPS1, DPM3, and RPS14P7 identified by VIS-NGS in the current retroviral screen and in the original tamoxifen-resistant clone VIII-18.
