# Supplementary material for: Identification of Resistance Genes in Breast Cancer Cells Treated with Fulvestrant and Ribociclib via Retroviral Screening and Integration Site Sequencing
Source: Cells. 2026 Jan 29;15(3):260. doi: 10.3390/cells15030260 (PMC12896645; doi:10.3390/cells15030260)
Supplement: Supplementary file 1 [file cells-15-00260-s001.zip › cells-4063176-supplementary.pdf]

Identification of Resistance Genes in Breast Cancer Cells Treated with Fulvestrant and Ribociclib via Retroviral Screening and Integration Site Sequencing

Zhangzan Huang [1], Corine Beaufort [1], Jean Helmijr [1], Brian Zantboer [1], Giada Rozema [1], Camilla Muritti [1], Julia J. Whien [1], Anna Uijterwegen [1], Michele Massimino [1,2,3], John W. M. Martens [1] and Maurice P. H. M. Jansen [1,*]

[1] Department of Medical Oncology, Erasmus MC Cancer Institute, University Medical Centre Rotterdam, Dr. Molewaterplein 40, 3015 GD Rotterdam, The Netherlands

[2] Department of General Surgery and Medical-Surgical Specialties, University of Catania, 95124 Catania, Italy

[3] Center of Experimental Oncology and Hematology, A.O.U. Policlinico "G. Rodolico—S. Marco", 95123 Catania, Italy

***** Correspondence: m.p.h.m.jansen@erasmusmc.nl

Supplemental Tables and Figures

Supplemental Table S1. Details of retroviral screen samples and VIS-NGS.

clone biological replicate	treatment type	treatment dose	treatment duration	Month library preparation/VIS-NGS	library_prep_ID	number detected VIS loci	Total RPM	median RPM	minimum RPM	maximum RPM
pLN1	untreated_NC	0	2 months	01/04/2024	RV_002	109	545369	1890	945	25047
pLN1	untreated_NC	0	3 months	01/05/2024	RV_015	41	326458	7112	1422	18492
pLN1	untreated_NC	0	1 month	01/03/2024	RV_018	66	269231	2051	1026	14359
pLN1	untreated_NC	0	1 month	01/03/2024	RV_021	37	166901	2817	1408	14085
pLN1	untreated_NC	0	4 months	01/06/2024	RV_061	66	430822	4795	1370	31507
pLN1	untreated_NC	0	1 month	01/09/2024	RV_069	41	583333	10204	3401	64626
pLN1	untreated_NC	0	2 months	01/10/2024	RV_083	108	385741	914	457	54388
pLN1	untreated_NC	0	2 months	01/10/2024	RV_084	63	525463	3086	1543	72531
pLN1	untreated_NC	0	1 month	01/01/2025	RV_092	34	519737	7237	2632	85526
pLN1	untreated_NC	0	1 month	01/01/2025	RV_110	75	291333	1333	667	31333
pLN2	untreated_NC	0	1 month	01/04/2024	RV_001	75	457460	2466	1233	30210
pLN2	untreated_NC	0	1 month	01/04/2024	RV_005	127	488083	2073	1036	15026
pLN2	untreated_NC	0	1 month	01/03/2024	RV_019	35	246744	5789	1447	20984
pLN2	untreated_NC	0	1 month	01/03/2024	RV_022	68	331539	3077	1538	33846
pLN2	untreated_NC	0	4 months	01/06/2024	RV_064	28	229000	5000	2000	30000
pLN2	untreated_NC	0	1 month	01/09/2024	RV_070	59	595238	3175	1587	99206
pLN2	untreated_NC	0	2 months	01/10/2024	RV_085	54	389535	3876	1292	54910
pLN2	untreated_NC	0	2 months	01/10/2024	RV_086	76	388737	2044	908	41326
pLN2	untreated_NC	0	1 month	01/01/2025	RV_098	35	416949	6780	3390	94915
pLN1	TAM	1000nM	2 months	01/04/2024	RV_003	91	495988	2006	1003	23069
pLN1	TAM	IC50	2 months	01/04/2024	RV_004	100	511723	2039	1019	23446
pLN1	TAM	IC50	3 months	01/05/2024	RV_016	24	274485	7088	2577	29639
pLN1	TAM	IC50	1 month	01/03/2024	RV_023	34	314721	7191	1692	21151
pLN2	TAM	1000nM	2 months	01/04/2024	RV_006	99	500000	2353	1176	25294
pLN2	TAM	IC50	2 months	01/04/2024	RV_007	71	402564	2051	1026	26667
pLN2	TAM	IC50	2 months	01/04/2024	RV_008	102	459270	1873	936	19195
pLN2	TAM	IC50	1 month	01/03/2024	RV_024	48	392157	4902	1961	27451
pLN1	FULV	1000nM	4 months	01/06/2024	RV_062	37	620028	8523	1420	76705
pLN1	FULV	1000nM	1 month	01/09/2024	RV_073	15	185529	3711	1855	91837
pLN1	FULV	1000nM	2 months	01/10/2024	RV_089	77	509901	2475	1238	90347
pLN1	FULV	1000nM	1 month	01/01/2025	RV_093	39	382434	2766	1383	71923
pLN1	FULV	1000nM	2 months	01/03/2025	RV_112	49	409330	2628	1314	34823
pLN1	FULV	1000nM	2 months	01/03/2025	RV_113	58	433308	1507	754	51997
pLN1	FULV	IC50	4 months	01/06/2024	RV_063	68	499400	3601	1200	42017
pLN1	FULV	IC50	1 month	01/09/2024	RV_077	34	307339	6116	3058	44343
pLN2	FULV	10000nM	2 months	01/03/2025	RV_115	60	462282	2530	920	41398
pLN2	FULV	1000nM	4 months	01/06/2024	RV_065	46	288019	4608	1536	15361
pLN2	FULV	1000nM	1 month	01/09/2024	RV_074	22	461374	8584	4292	120172
pLN2	FULV	1000nM	2 months	01/10/2024	RV_090	56	508050	3578	1789	59034
pLN2	FULV	1000nM	1 month	01/01/2025	RV_099	51	478434	4792	1597	74281
pLN2	FULV	IC50	months	01/06/2024	RV_066	40	195467	2833	1416	18414
pLN2	FULV	IC50	1 month	01/09/2024	RV_078	34	347368	7018	3509	82456
pLN1	RIBO	1000nM	1 month	01/01/2025	RV_094	41	361765	5882	2941	44118
pLN1	RIBO	1000nM	2 months	01/03/2025	RV_118	80	321283	1364	682	55935
pLN1	RIBO	1000nM	2 months	01/03/2025	RV_121	56	387078	2328	1164	64610
pLN2	RIBO	10000nM	2 months	01/03/2025	RV_123	45	400320	2802	801	50841
pLN2	RIBO	1000nM	1 month	01/01/2025	RV_100	40	349143	3429	1143	57143
pLN2	RIBO	1000nM	2 months	01/03/2025	RV_119	47	369458	2463	1232	57882
pLN2	RIBO	1000nM	2 months	01/03/2025	RV_122	82	329208	1238	619	36510
pLN1	ABEMA	1000nM	1 month	01/01/2025	RV_096	31	333051	5932	1695	44068
pLN1	ABEMA	1000nM	2 months	01/03/2025	RV_127	48	414420	4689	1172	101407
pLN2	ABEMA	1000nM	1 month	01/01/2025	RV_102	68	454128	3058	1529	32110
pLN2	ABEMA	1000nM	2 months	01/03/2025	RV_128	60	347237	2167	1083	32503
pLN1	PALBO	1000nM	1 month	01/01/2025	RV_095	29	398108	2911	1456	107715
pLN1	PALBO	1000nM	2 months	01/03/2025	RV_125	79	273624	1360	680	31271
pLN2	PALBO	10000nM	2 months	01/03/2025	RV_126	89	256785	1392	696	19137
pLN2	PALBO	1000nM	1 month	01/01/2025	RV_101	61	466601	3929	1965	91356
pLN1	FULV/RIBO	1000nM	1 month	01/01/2025	RV_097	67	370596	2710	1355	44715
pLN1	FULV/RIBO	1000nM	2 months	01/03/2025	RV_120	25	591185	6079	3040	103344
pLN2	FULV/RIBO	1000nM	1 month	01/01/2025	RV_103	33	439394	7576	3788	51136
					**Average:**	**57**	**398170**	**3863**	**1584**	**49486**

Supplemental Table S2. Compound details.

Compound		Company	Category number	Stock concentration
Endocrine therapy	Tamoxifen	MedChemExpress	HY-16950	64.5nM
	Fulvestrant	MedChemExpress	HY-13636	82.4nM
CDK4/6 inhibitors	Ribociclib	Selleckchem	S7440	23.0nM
	Abemaciclib	Selleckchem	S5716	12.3nM
	Palbociclib	Bio-Connect	827022-33-3	174.3nM

Supplementary Table S3. Details of 3902 VIS loci detected in retroviral screen.

This table is added as excel file with multiple sheets.

The sheet 3902 VIS contains the following columns: gene_name, gene_strand, gene_width, distance_to_gene, left_coverage, right_coverage, orientation, coordinates, identical coordinates, 218 VIS-LIST, 89 VIS-LIST, 16VIS-LIST, 37VIS-LIST, sample_count, and for all 60 samples the RPM levels measured for each of 3902 VIS loci. The end of the sheet summarizes for each locus in how many samples it was detected in total, and for pLN1 and pLN2 only, and in total for each treatment condition the detection frequency and average read depth coverag.

The identical coordinates column can be used to select unique VIS loci by blanks and VIS locus multiple links_1 and excluding links_2-4 for each of the mentioned locus in the VIS-LIST columns, because some VIS loci are linked to multiple genes. The VIS-LISTs columns can be used to extract detection frequencies and average read coverage

Supplementary Table S4. Details and Analysis summary of 218 VIS loci detected in both biological replicates.

This table is also in the excel file. The sheet 218 VIS loci and contains next to RPMs for each of the 218 VIS loci also analyses details including p-values of the Fisher Exact test and the detection frequencies in untreated (NC, negative controls) pLN1 and pLN2, and treated pLN1 and pLN2 for each of the treatments. It contains also fold changes for each treatment compared to untreated.

Supplemental Table S5.Candidate resistance VIS loci (*n*=89) for tamoxifen, fulvestrant, and/or ribociclib with diferential detection frequencies compared to controls (Fisher Exact p-value <0.1).

Supplemental Table S6. Candidate resistance VIS loci (*n*=16) for tamoxifen, fulvestrant, and/or ribociclib with differential read coverage compared to controls (Students T-test p-value <0.1).

Supplemental Table S7. VIS-locus coordinates of *TRPS1, DPM3,* and *RPS14P7* identified by VIS-NGS in the current retroviral screen and in the original tamoxifen-resistant clone VIII-18.

	**VIS-locus coordinates identified by VIS-NGS**	
**Gene**	**Retroviral screen**	**Tamoxifen-resistant clone VIII-18**
TRPS1	chr8:115688896-115689615	chr8:115688893-115689408
DPM3	chr1:155139443-155140451	chr1:155140064-155140451
RPS14P7	chr4:161020234-161020837	chr4:161020232-161020577

Supplementary Figure S1

Details of VIS loci related to fulvestrant and ribociclib resistance			Detection frequencies							Average Read depth coverages						
			Discovery		Verification					Discovery		Verification				
Gene	VIS coordinates	VIS distance to gene	NC	Tamoxifen	Fulvestrant	Ribociclib	Abemaciclib*	Palbociclib*	Fulvestrant/Ribociclib*	NC	Tamoxifen	Fulvestrant	Ribociclib	Abemaciclib*	Palbociclib*	Fulvestrant/Ribociclib*
BCAR3	chr1:93583745-93584311	0	37%	25%	60%	71%	25%	25%	33%	19244	1941	12875	12960	1083	10806	24621
CCND1	chr11:69637615-69638759	2397	16%	63%	7%	0%	0%	0%	0%	5272	2337	4249	0	0	0	0
CHL1	chr3:262625-262936	0	26%	88%	33%	0%	0%	0%	0%	1589	8307	29742	0	0	0	0
EGFR	chr7:54994941-54995270	23747	16%	63%	20%	0%	0%	0%	0%	2191	10902	22085	0	0	0	0
			1.00	1.00	1.00	1.00	1.00	1.00	1.00	10000	10000	10000	10000	10000	10000	10000

were only detected in one of both biological replicates

Supplementary Figure S1. This figure presents the findings for 4 candidate resistance genes associated with VIS loci that are enriched in tamoxifen-resistant cells. It shows the detection frequencies and average read depth coverage for all monotherapy treatments and the fulvestrant and ribociclib combination therapy.
